# Analysis the characterization of climate change and its impacts on smallholder farmers in Eastern Ethiopia

**DOI:** 10.1016/j.heliyon.2023.e20293

**Published:** 2023-09-21

**Authors:** Girma Asefa Bogale

**Affiliations:** School of Natural Resources Management and Environmental Sciences, College of Agriculture and Environmental Sciences, Haramaya University, P.O. Box 138, Diredawa, Ethiopia

**Keywords:** Climate change, Drought stress, Soil moisture stress, Trends analysis, LGS

## Abstract

The impact of climate change on agriculture and food production is manifested through alterations in agro-ecological factors. The region of sub-Saharan Africa is particularly susceptible to the effects of climate change and variability, given its reliance on rainfall-dependent agriculture and inadequate adaptive capabilities. The objective of this study was to analysis of the characterization of climate change and its impacts on smallholder farmers in eastern Ethiopia. Historical daily rainfall, maximum and minimum temperature in 1991–2021 years of 12 weather station were collected from National Meteorological Institute of Ethiopia and NASA/POWER. Data quality control, trend test and outlier detection test were used. Climate characterization (onset, cessation, LGS and Dry/wet spell length) and precipitation concentration index were examined. The earliest, and latest start of rainy season in Kombolcha and Haramaya were 81DOY (March 21) and 225DOY (August 13) within mean 130DOY and 125DOY, respectively. In study area the minimum and maximum length of growing period of was recorded in Chinakesen and Haramaya by the values of 32DOY and 253DOY (Days of the year) respectively. In this arid and semi-arid areas, growing period was recorded under the short season crop maturity while long cropping season maturity does no satisfy crop water requirement due to moisture stress. The coefficient of variation of length of growing season from Mieso and Chinakesen were 36.2% and 47.9% which implies that the areas were highly vulnerable to climate extreme events of drought. Length of growing season (LGS) of Gemechis district was decreasing by a factor −2.533 shows highly significant at alpha value of 0.05 confidence level. Dry spell length probability occurrence of 5 days during the main cropping rainy season is significantly very high. The 30 years of data record suggests that a 29–48% precipitation concentration index of kiremt (JJAS) and belg (FMAM) seasonal rainfall which are demonstrating irregular precipitation. kiremt (JJAS) rainfall trend tests of Babile, Kurfa chale and Mieso districts were increasing by a factor of 2.016, 2.043 and 2.117, respectively and statistically significant at 95% confidence level, in the time period of 1991–2021 years. If furtherstudy will be examined climate extreme indices and adaptation strategies designed to simulate the impacts and variability of climate change in the study area.

## 1 Introduction

Climate change is one of the greatest challenges of humankind in the 21st century which indicates that the earth's climate is rapidly changing, owing to increase in greenhouse gas emissions [1,2]. Another study by Ref. [3] revealed that, the global average global temperature shown in a warming of 0.78 °C over the period of 1850–2012 and future climate projection increase between 1.5 and 2 °C at the end of the 21st century. The increased concentration of greenhouse gases has raised the average temperature and altered the amount and distribution of rainfall globally [4,5]. Agriculture is highly vulnerable to climatic and environmental changes [6]. According to the Intergovernmental Panel on Climate Change (IPCC) [AR6], Africa is one the most vulnerable continents to climate change and climate change [7,8]. The El Niño- Southern Oscillation (ENSO) through its warm (El Nino) and cold (La Niña) phases causes variability of seasonal rainfall leading to drought and flood at extreme case [9]. In Africa, precipitation variability is expected to increase, while precipitation is likely to decrease over most regions of sub-Saharan Africa (SSA) [10]. However, this system in sub-Saharan Africa is a risky proposition due to its low and irregular rainfall and exposure to long periods of drought [11].

The seasonal and interannual variability of rainfall in certain regions influences the management rainfall for various anthropogenic uses [12,13]. Rainfed agricultural practices significantly impact the economy of developing countries; however, these practices are highly vulnerable to the effects of weather and climate [14,15]. This is partly due to a high reliance on rain-fed agriculture, low adaptive capacity, and an increasing reliance on natural resources for livelihoods [[16], [17], [18]]. Precipitation and temperature are two of the most important variables in the fields of climate science and hydrology and are often used to track the extent and magnitude of climate variability and variability (IPCC, 2007, [7,8]. According to the findings of [19] found that in countries whose economies rely heavily on low-productivity rain-fed agriculture, rainfall trends and variability are often the factors that explain various socioeconomic problems such as food insecurity emphasis on being quoted.

Global warming is intensifying the global hydrological cycle, making the wet and dry seasons more contrasting and more intense, while the frequency of sub seasonal precipitation is decreasing [[20], [21], [22]]. Rainy/dry periods or consecutive periods of rainy/dry days can be used to describe local weather patterns [23]. The uneven seasonal distribution of rainfall can expose crops to a range of intra-seasonal droughts that can affect yields, ranging from mild to severe [24]. Large anomalies in the occurrence and duration of wet and dry seasons can have significant impacts on extreme hydrological events, vegetation growth and food production [22,23,25,26]. Characterizing the occurrence and causes of rainy/drought periods is therefore central to understanding, predicting extreme hydrological events, and managing climate-related risks [27]. The intensity [28], frequency [29], and pattern [30] of precipitation are expected to change, which can cause change in evaporation and temeperature [31], and more extreme weather events, such as floods [32] and droughts [33], are more likely to occur alternately. Thus, higher temperatures lead to increased evaporation and extreme drought, while greater atmospheric water storage capacity can lead to increased precipitation [[34], [35], [36]].

An increase in mild and extreme events has been emphasized at the expense of dry and warm events [37]. Additionally, prolonged dry seasons and wet weather (periods) can have a significant impact on extreme hydrology. The wet season (WS) is typically a series of wet days (WD), i.e. days with more than 1 mm of precipitation, followed by at least one dry day (DD) (i.e. with less than 1 mm of precipitation). Defined as the following day [38,39]. Dry season (DS) is similarly defined as a sequence of consecutive DDs preceded and followed by WS. Precipitation directly affects the availability of water resources and is one of the most important climatic and hydrological parameters [40]. The variation of precipitation concentration is critical to water resource utilization and it affects human life, environment and ecosystem. For instance, the changes of precipitation concentration is important to adjust the optimal allocation scheme of soil and water resources in time and to ensure higher yields of crops [41]. In addition, this kind of precipitation data can be used to understand the response of the water cycle to climate change, and thus its impact on water resource availability at regional and global scales [[42], [43], [44]].

The precipitation concentration index (PCI) is a powerful indicator for temporal precipitation distribution and is also very useful for the assessment of seasonal precipitation changes. Moreover, investgating the temporal-spatial variations of precipitation in previous time periods is a critical for making reliable predictions of future climate changes and it has been used to describe climate of a particular place or region [45]. Furthermore, precipitation concentration over time is an important parameter for monitoring the rate of physical processes in the atmosphere [46]. Studying local climate characterization of early onset of the rainy season leads to crop germination, since most farmers sow in dry soil. If a dry spell follows. The seedlings die a “false start” [[47], [48], [49]] and often lead to be reshown. According to (Z. T. and P. J. L [50]. revealed that, the major causes of agricultural failure in rift valley of Ethiopia are frequent dry spells of about 10 days length, as well as a shorter growing period due to replanting or late onset and/or early cessation of rain. Reliable estimation of onset, ceasstion of rain and length of growing season could help optimize agricultural productivity strategies in semi-arid areas [51].

Climate change is a global phenomenon that has far-reaching consequences, particularly for vulnerable commuinities relying on agriculture for their livelihoods. Many agricultural activities and sustainable food production planning rely on rainfall for land preparation, seed/crop planting and harvesting, so the onset, end, and length of the growing season which are required and escaped crop from drought. It is of utmost importance to know and change inequality nationally and internationally [52]. It has been observed that over the years the erratic start and end of the rainy season in many areas has made it difficult for farmers to optimize sowing timing and adapt to the length of the growing season [53]. It is a problem for farmers in terms of optimizing seed sowing and the need to adapt to the length of the growing season [51,54]. Eastern Ethiopia, known for its predominantly agrarian economy, is highly susceptible to the adverse effects of climate change. This study explores the specific challenges faced by smallholder farmers in the study area and examines the implications for their agricultural practices, food security and overall well being. In this situation of pronounced seasonal precipitation, the variability of rainfall onset and stoppage is of great importance and requires its estimation and forecasting [47]. Validation of satellite-generated climate data is a necessary step to support agricultural research and development [55]. Climate change is a pressing global issue with significant implications for agricultural-dependent regions like Eastern Ethiopia [56]. A study conducted by Ref. [57] showed that high temperature and rainfall variability significantly influenced the agriculture sector in Ethiopia. Specifically, agricultural production of Kurfa Chele district is frequently affected by adverse effects of climate related shocks [58]. Therefore, the aim of this research article was to analysis the characterization of climate change and its impacts on smallholder farmers in east and west hararghe zone, eastern Ethiopia.

## 2 Materials and methods

### 2.1 Description of the study area

**Fig. 1:**
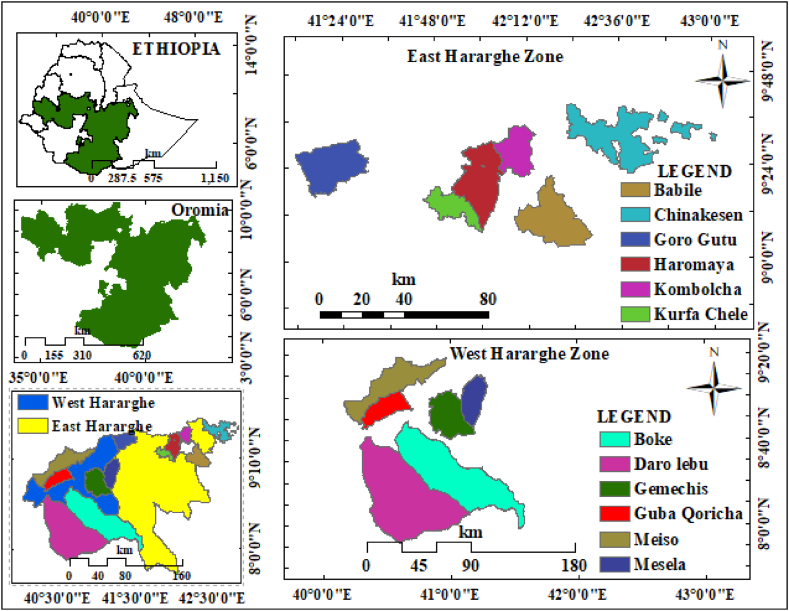
Map of the weather station districts of eastern and western hararghe zone.

The study was conducted East and West Hararghe zone (selected 12 weather stations of the districts) Oromia Region, Eastern Ethiopia (Fig. 1). Mean annual precipitation, maximum and minimum temperatures of 790 mm/yr, 24.7 °C and 11.5 °C, respectively, are observed over an altitude range of 1200–2460 m above sea level, especially in the eastern hararghe zone (Nigussie et al., 2018). The region receives bimodal rainfall, including the belg rainy season from February to May and the kiremt rainy season from June to September. Similarly, in the western hararghe zone, the mean annual variability of temperature and precipitation was 21 °C and 945 mm at elevations of 1107–3106 m. a.s.l. Most of the smallholder farmers live in idyllic settlements, but some grow sesame seeds for the market in the hararghe zone, and other crops such as sorghum, maize and green beans for food. Major soil types in the study area include leptosol, cambisol, rubisol, fluvisol, vertisol, nitisol, and regosol [59]. Agricultural systems are mixed systems of crops and livestock. Major crops grown include sorghum, maize, legumes, wheat, oilseeds, vegetables, fruits, and cash crops such as coffee (Coffee arabica L.) and khat (Catha edulis forsk) [60].

### 2.2 Datasets and sorces

**Fig. 2:**
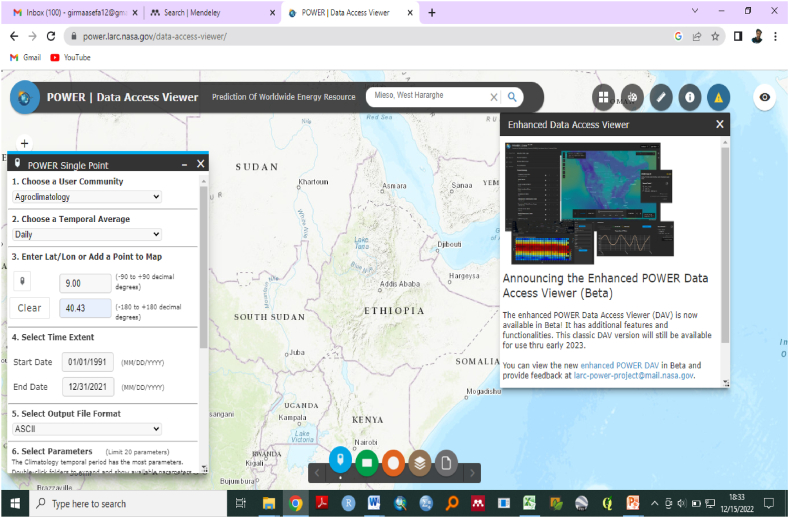
Grided climate data of the study area.

In this regards, historical data on daily precipitation, maximum and minimum temperature from 1991 to 2021 were collected from National Meteorolgical Institute of Ethiopia and National Aeronautics and Space Administration/Prediction Global Energy Resources (NASA/POWER) (https://power.larc.nasa.gov/dataset [61]. The project contains over 380 satellite-based meteorological and solar energy analysis (ARD) data on four timescales. POWER Data Archive provides single point global data at the native resolution of the source data product and 0.5 × 0.5-degree resolution for regional and global data requests. An improved POWER Data Access Viewer (DAV) is now available (Fig. 2).

### 2.3 Data analysis

#### 2.3.1 Data quality control

Climatic data is very sensitive to irregular or outlier values as it can affect the quality of the data. Therefore, before analysing time-series climate data, systematic outlier trimming is essential to reduce the tail size of the distribution and to obtain reproducible and reliable results [62,63]. In this study, the Tukey fence as outlined was used to detect outliers through the XLSTAT2014 software [64]. The data range is represented by formula Equation (1).

(1) [Q1−1.5*IQR,Q3+1.5*IQR]

where Q1 and Q3 are the lower and upper quartile points respectively, 1.5 is standard deviation from the mean, and IQR is the interquartile range. Values outside the Tukey fence are considered as outliers. In this study, such outliers were set to a limit value corresponding to ±1.5 × IQR.

Then, a standard outlier threshold, which is defined using a parameter of inter-quartile range (IQR), was used as described in Ref. [63] as following Equation (2);

(2) Threshold=Q3+(3*IQR)

where Q3 is third quartile and IQR is an inter-quartile range. The inter-quartile range method is known as a technique which is not highly sensitive to outliers but still keeps the information of extremes [63]. The detected outlier values were then removed and substituted by outlier threshold as indicated.

#### 2.3.2 Climate characterization

In this study, onset and cessation of the rainy season was determined by the accumulation of a total of 20 mm of rainfall over 3 consecutive days, followed by a dry period not exceeding 10 days over the next 30 days. The day after decade in the first of September, the soil water balance reached zero [65,66]. Set-up day required day 4 mm/day ETo and 100 mm/m actual soil water holding capacity (at field volume) were calculated using the statistical software InStat plus (v3.36) and CROPWAT_v8.0 respectively [66]. Length growing season (LGS) is an important factor in determining the maturity of cultivars grown under different rainfall conditions [67]. LGS was calculated by subtracting the rainy season start date from the growing season end date [68]. The length of the dry season in the kiremt season was calculated using a first-order Markov analysis [66]. Drying period lengths over 5, 7, 10 and 15 days were analysed with the software InStat (+v3.36) [66].

#### 2.3.3 Variability and trend analysis

Long term data of rainfall and temperature, which generally fall into variability (coefficient of variation (CV %)) and trend (MZ) were employed by XLSTAT2018 version statistical software. Trend detection and analysis are performed through parametric and non-parametric tests only for consistent data. The advantage of non-parametric statistical test over the parametric test is that the former is more suitable for non-normally distributed, outlier, censored and missing data, which are frequently encountered in hydrological time series. The nonparametric Mann-Kendall (MZ) test was used to characterize the trends of the precipitation concentration index (PCI), onset, cessation date, and length of growing period/season crops, minimum and maximum temperatures in this study. This test is widely used to analyze the monotonically increasing or decreasing trends in climate change research [[69], [70], [71], [72], [73]]. Coefficient of variation (CV%) is calculated to evaluate the variability of rainfall and temperature of the study area as computed in Equation (3),

(3) $$CV=\frac{\delta }{\mu }x100$$

where: σ, standard deviation; μ, mean Where CV is coefficient of variation, S is the standard deviation and $\bar{x}$ is mean for rainfall. When CV<20% is less variable data, CV from 20% to 30% is moderate variable and CV>30% is defined as highly variable. Areas with CV>30% said to be vulnerable drought [74].

MK test is a non-parameters test, which tests for a trend in a time series without specifying whether the trend is linear or non-linear [75]. MK trend test is a non-parametric test commonly employed to detect monotonic trends in series of environmental data, climate data or hydrological data. MK test has been used to detect the presence of monotonic (increasing or decreasing) trends in the study area and whether the trend is statistically significant or not. The ZM test statistic “S” is calculated based on [[75], [76], [77]] using the formula in Equation (4);

(4) $$S=\sum_{i=1}^{n-1}\sum_{j=i+1}^{n}sgs\left(xj-xi\right)$$

The application of trend test is done to time series X_1_ that is ranked from i = 1, 2 … n-1 and X_j_, which is ranked from j = i+1, 2 … n. Each of the data point Xi is taken as a reference point which is compared with the rest of the data points X_j_ so that Equation (5);

(5) $$sgs\;\left(xj-xi\right)\left\{ \begin{array}{c} +\text{lif}\left(\text{xj}-\text{xi}\right)>0\\ 0\text{if}\left(\text{xj}-\text{xi}\right)=0\\ -\text{lif}\left(\text{xj}-\text{xi}\right)<0 \end{array}\right.$$

where x _i_ and x_j_ are the annual values in years i and j (j > i) respectively. It has been interpreted that when the number of observation is more than 10 (n ≥ 10), the statistic ‘S’ is approximately normally distributed with the mean and E(S) becomes 0 [76]. In this regard, the variance statistic is given in Equation (6):

(6) $$Var\;\left(S\right)=\frac{n\left(n-1\right)\left(2n+5\right)-\sum_{t=1}^{m}f1\left(t_{1}-1\right)\left(2t_{1}+5\right)}{18}$$

where n is the number of observation and t_i_ are the ties of the sample time series. The test statistics Z_c_ is as follows in Equation (7):

(7) $$Z=\left\{ \begin{array}{c} \frac{s-1}{\delta }ifs>0\\ 0ifs=0\\ \frac{s+1}{\delta }ifs<0 \end{array}\right.$$

where Z_c_ follows a normal distribution, a positive Z_c_ and negative Z_c_ depict an upward and downwards trend for the period respectively. A significance level α is also utilized for testing either an upward or downward monotone trend (a two-tailed test). If Zc appears greater than Z α/2 where α depicts the significance level, then the trend is considered as significant.

Sen's Slope estimation test computes both the slope (i.e., the linear rate of change) and intercept according to Sen's method. The magnitude of the trend is predicted and slope estimator methods [78,79]. A positive value of β indicates an ‘upward trend’ (increasing values with time); while a negative value of β indicates a ‘downward trend’. Here, the slope (T_i_) of all data pairs is computed as [78]. In general, the slope T_i_ between any two values of a time series x can be estimated in Equation (8):

(8) $$T_{i}=\frac{x_{j}-x_{i}}{j-k}$$

where x_j_ and x_k_ are considered as data values at time j and k (j > i) correspondingly. The median of these N values of T_i_ is represented as Sen's estimator of slope which is computed as Q_med_ = T_(N+2)/2_ if N appears odd, and it is considered as Q_med_ = [TN/2 + T(_(N+2)/2)/_2] if N appears even. A positive value of Q_i_ indicates an upward or increasing trend and a negative value of Qi gives a downward or decreasing trend in the time series.

#### 2.3.4 Precipitation concentration index (PCI)

In this study**,** the precipitation concentration index (PCI) proposed by Ref. [80] and developed by Ref. [81] was employed by R_studio program software version 2021 for the calculation of annual PCI, as indicated in Equation (9):

(9) $$PCI=\frac{\sum_{i=1}^{12}Pi^{2}}{{\left(\sum_{i=1}^{12}Pi\right)}^{2}}x100$$

where *pi* represents the precipitation in month *i* that is calculated for each studied station and for each year throughout the observation period. In addition, the PCI was calculated for each studied on seasonal scale (i.e., SPCI) for *kiremt* (JJAS), and *belg* (FMAM) and bega (ONDJ), according to equation (10):

(10) $$SPCI=\frac{\sum_{i=1}^{4}Pi^{2}}{{\left(\sum_{i=1}^{4}Pi\right)}^{2}}x100$$

PCI or SPCI values below denote uniform monthly rainfall distribution throughout the year (low precipitation concentration); values ranging from 11 to 15 indicate a moderate concentration of precipitation; values between 16 and 20 represent an irregular distribution; and values above 20 represent a strong irregularity (high precipitation concentration) in precipitation distribution [44,80].

#### 2.3.5 Standardized rainfall anomaly index (SRAI)

Annual precipitation data in east and west districts of hararghe zone were investigated using SRAI which has grown in popularity for regional climate change studies [82]. The dry years and wet years within a span of time [83] can be found in the following equation (11):

(11) $$\text{SRAI}=\frac{X_{i}-\bar{X}}{\delta }$$

where $X_{i}$, and $\bar{X}$ are the annual and seasonal rainfall of the particular year and the long-term mean annual rainfall over a given period of observation. The negative values present a drought while the positive values present a wet episode [84]. proposed the categorization of the SRAI values and presented in Table 1.

**Table 1:** Standardized rainfall anomaly index classification [85].

Rainfall anomaly index range	Class description
≥3.0	Extremely wet
2.00 to 2.99	Very wet
1.00 to 1.99	Moderately wet
0.50 to 0.99	Slightly wet
0.49 to −0.49	Near normal
−0.50 to −0.99	Slightly dry
−1.00 to −1.99	Moderately dry
−2.00 to −0.99	Very dry
≤-3.00	Extremely dry

Rainfall anomaly index is dimensionless and it has also been used to determine variation in rainfall for different agroecological zones of in east and west districts of hararghe zone. Rainfall anomaly index classification is used to determine extremes condition of rainfall in a particular area for decadal and annual time frames (Nyuyfoni, 2021). The results gotten from the range and class description determine the variation in rainfall for that region which may be negative or positive. This range is from ≥3.00 (extremely wet) to ≤3.0 (extremely dry) (Table 1).

## 3 Results

### 3.1 Climate characterization of hararghe zone

Characterization of climate features are interesting in understanding seasonal, and annual patterns of rainfall by investigating variables such as amount of rainfall, rainy days, length of growing season and frequency of dry spells. Another study (Seleshi, 2004 [[86], [87], [88]]; characterized annual and seasonal rainfall totals and rainy days in Ethiopia and the Sudano-Sahelian, respectively, and all the cases have exhibited high variability. Table 2 shows the characterization of rainfall onset, cessation date and length of the growing season for the specific study area. In this study, the minimum and maximum or the earliest and latest start (onset date) of the annual rainy season in Kombolcha and Haramaya were 81 DOY (21 March) and 225 DOY (13 August), respectively, recorded in Table 2. This results was congruent with the findings of [47,52,89,90] the onset dates and cessation dates of the rainy season are critical rainfall attributes that relate to the effective rainfall which is more important to cropping season than the total annual rainfall.

**Table 2:** Descriptive statistics of rainfall characteristics in the study area from 1991 to 2021 years.

Districts	SOS Min	max	EOS min	max	LGP min	max	Q25% SOS EOS LGP	Q75% SOS EOS LGP	Mean SOS EOS LGP	CV% SOS EOS LGP	ZK SOS EOS LGP	Slope SOS EOS LGP
Babile	91	224	245	305	37	145	93	117	106	33.1	0.184	0.417
							245	249	252	5.6	0.226	0
							132	158	1445	21.5	−0.096	−0.308
Chinakesen	106	231	248	337	32	166	109	202	156	29.4	0.172	1.857
							248	298	283	9.3	−0.123	−0.286
							81	187	126	47.9	−0.224	−2.533
Kombolcha	81	225	257	303	40	239	92	190	130	38.1	0.219	0.727
							257	258	260	3.6	0.195	0
							109	169	131	38.4	−0.118	−0.571
Haramaya	81	225	259	338	47	253	92	148	125	38.6	0.167	0.625
							276	304	288	7.0	0.035	0.095
							124	201	163	33.8	−0.145	−0.889
Goro gutu	88	225	273	291	48	199	94	190	130	37.4	0.206	0.667
							273	273	273	1.2	0.330	0
							85	179	143	34.5	−0.180	−0.6
Kurfa chale	92	211	246	308	58	174	93	117	108	22.9	0.131	0.136
							246	247	250	4.9	0.259	0
							133	154	141	15.1	−0.050	0.071
Mieso	103	2017	250	313	33	188	106	167	133	29.0	−0.037	0
							250	259	260	7.4	0.143	0
							83	147	127	36.2	0.093	0.375
Guba qoricha	107	217	250	315	49	202	108	179	140	27.5	0.059	0.083
							258	293	278	7.1	0.120	0.333
							103	174	138	31.7	0.063	0.267
Boke	92	211	245	309	41	197	93	129	118	29.0	0.017	0
							245	279	149	23.9	0.167	0.4
							136	176	149	23.4	0.106	0.4
Gemechis	92	217	246	296	45	193	93	118	116	30.7	0.012	0
							246	250	251	4.4	−0.211	0
							128	154	134.9	27.9	−0.075	−0.111
Mesela	103	217	250	313	52	194	106	167	133	29.02	0.035	0
							250	259	260	7.4	0.164	0.375
							140	179	153	25.7	0.125	0.44
Daro lebu	92	224	245	339	44	247	103	183	136	32.6	0.007	0
							245	299	277	10.6	−0.050	0
							109	177	141	35.3	0.028	0

SOS, Start of rainy season: EOS, End of rainy season: LGP, Length growing period: Quartile 25%: Quartile 75%: CV%, Coefficient variation; ZMK; Mann–Kendall trend test, slope (Sen's slope) is change/yr, and *, **, significant at 0.05 significance level.

The previous study revealed that, the knowlgde of the dates of the onset and the cessation of the rainy season is critical to crop production during flowering and maturity stage especially in sub-Saharan Africa [[91], [92], [93]]. Furthermore, the minimum value at the end of the rainy season (end date) for Babile, Boke and Daro lebu was 245 DOY (September 2nd), while the maximum value of 339 DOY (December 5th) was 252 observed within 149, and 277 DOY. The impact of climate change on Ethiopian agriculture has been found to be very strong. Climate change plays an important role in agricultural production, significantly affecting plant growth development and yields, making agricultural activity one of the most sensitive and vulnerable sectors of human activity (Ventrella et al., 2012). Regarding to CV% values except Boke district, wherein the CV% cost became 23.9% and there has been a moderate however discernible volatility withinside the place, the coefficients of the cease of the wet season had been additionally much less variable in all of the station regions analysed. The results were in line with the findings of [19]; Y. and U. Z [94]. (diagnosed that valuable rift valley stations have insignificant variability of annual rainfall whilst there has been massive seasonal variability even for the duration of the main wet season.

The coefficients of variation for LGSs from Mieso and Chinakesen were 36.2% and 47.9%, indicating that the region was highly vulnerable to extreme weather events such as drought. The results indicated that water availability is the main time-limiting constraint for rain-fed agriculture in arid and semi-arid regions such as Gedarifu province, Sudan [95]; Abiodun BJ, 2016 [96]; were consistent. This is where plants can grow, and inconsistencies in the reliability and distribution of rainfall contribute significantly to reduced yields and large inter-annual variations in production. This is because when LGS changes, the soil becomes less fertile and lacks the nutrients that plants need, and the tranquillity it gains is the potential for future growth of non-growing raw materials or microorganisms that evaporate from it due to heat causing moisture stress. According to (Isabella et al., 2001), drought is one of the extreme climate events that impairs the economic and social values of societies.

The Mann-Kendall nonparametric test shows that the 31-year precipitation trends from January to December are correlated with the slope magnitude of Sen (Q) at the beginning, end, and length of the growing season for each district was calculated (Table 2). In this result, the Mann-Kendall test for the start date for all districts increased slightly, but Mieso's Zc value was −0.037, indicating a negative trend, and the alpha value for all districts increasing and non-significant statistically (Table 1). Cessation dates for Chinakesen, Gemechis and Daro lebu districts have decreased by a factor of −0.123, −0.211 and −0.050 respectively over the past 30 years, showing a negative trend representing a state of little importance (Table 2). The Mann-Kendall test of the LGP for Babile, Chinakesen, Kombolcha, Haramaya, Gorogutu, Kurfachale and Gemechis districts showed a decreasing trend, which was not significant at an alpha value of 0.05 confidence level.

### 3.2 The probability of dry spell length occurrence in the districts of hararghe zone

**Fig. 3:**
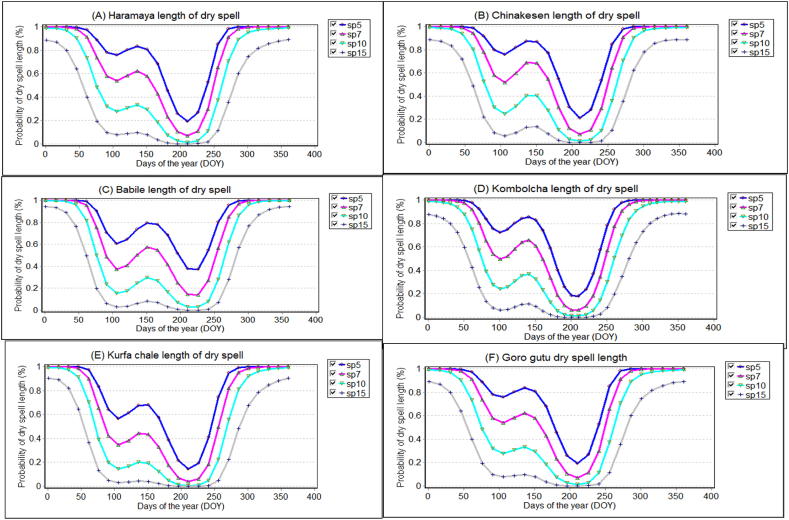
Dry spell length at 5, 7, 10 and 15 days for selected districts of east hararghe zone in 1991–2021 years in the study area.

**Fig. 4:**
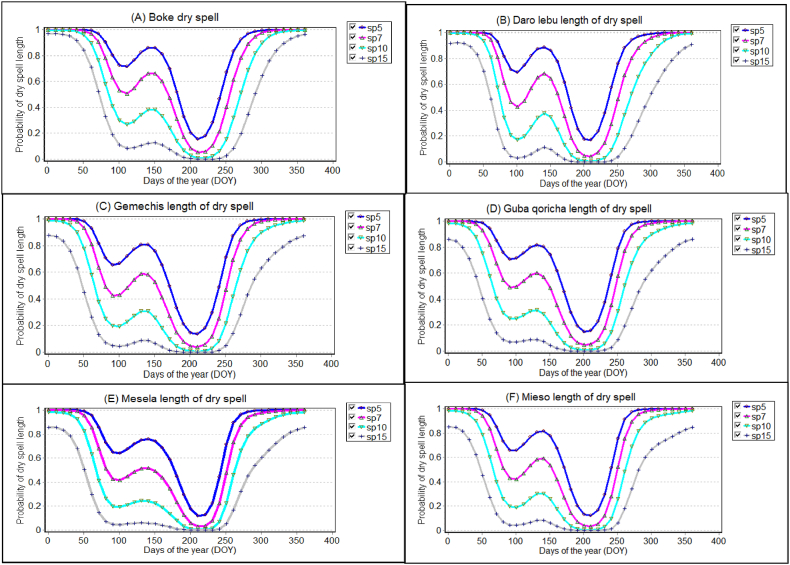
Probability of dry spell length at 5, 7, 10- and 15-day's occurrence for selected districts of west hararghe zone from 1991 to 2021 years.

The probabilities of dry season length after 5, 7, 10, and 15 days were characterized for each district in the East Hararghe Zone (Fig. 3, Fig. 4). In the main rainy season, there was a likelihood of more than 60% for a 7-day period of dry weather. This probability increased significantly, almost reaching 100%, in districts such as Chinakesen, Kombolcha, and Goro gutu (Fig. 3). Similarly, during the same season, the probability of a 7-day dry spell exceeding 60% was nearly 100% in districts including, Daro lebu, Guba Koricha, and Mieso (Fig. 4). As the length of dry spells increases, human health is impacted by disease, famine, and food insecurity. Crop production declines due to insufficient rainfall during crucial growth stages, leading to challenges and economic decline, particularly in Ethiopia's arid and semiarid regions. This study was confirmed by Refs. [97,98] shows the relationship between climate change and migration in Africa, the continent with the fastest projected population growth is one of the most vulnerable to climate change due to its high exposure and low adaptative capacity. Drought period lengths of 10 and 15 days were associated with 20% and 40%, respectively, of the probability of a drought period occurring, whereas the 10-day probability in the Kurfa Chale district was less than 20% of the drought period (Fig. 3). Drought during the main rainy season. Previous studies have also investigated within-season dry spell and their impact on planting dates and crop yield [[99], [100], [101], [102]]. The probability of 5, 7, 10, and 15 occurring in the next 30 days is very high in January, February, March, and April, and most likely in May, June, July, August, and September.

Furthermore, in the western Hararghe zone, specifically at six selected stations (Fig. 4), the likelihood of experiencing longer dry spells lasting more than 15 days was around 20% in April. This probability then decreased steadily from the middle to the end of June, reaching zero. Subsequently, it increased again after the end of August (Fig. 4). For all stations in the east and west Hararghe zones of the Oromia region, the probability of 5-day and 7-day rainfall was at its peak point during the months of July and August. Notably, all the probability curves for dry spells converged to their minimum levels during the peak rainy season, which occurs between the 185th and 240th day of the year. They then decreased again around September (245th to 270th day of the year), indicating the end of the growing season, particularly in the Mieso and Boke districts of the western Hararghe zone. These areas faced dry conditions, making crop production challenging and vulnerable to drought (Fig. 4). In Mieso district specifically, smallholder farmers frequently practice pastoralism, and the local population often experiences food security issues due to extreme weather events such as droughts and floods. Moreover, due to limited availability of land, when dry spells extended beyond a certain length, the yields of short and long maturity crops were severely reduced throughout the year.

### 3.3 Precipitation concentration index (PCI)

Precipitation concentration index is a useful for exploring the risks related to extreme precipitation events. Furthermore, the precipitation concentration is remarkably useful in the context of climate change, because it is able to assess the repercussions of a water cycle enforcing due to an increase in evapotranspiration especially the high-intensity of precipitation [103,104]. Precipitation concentration index (PCI%) were variable, varying primarily from 13 to 16, 29 to 30, and 37–48%, respectively, for the station areas shown in Table 3. In this found that the lowest annual PCI values occurred near Boke 13.56% and Gemechis 13.78%. This represents a reasonably uniform seasonal variation in precipitation in the study area. As the result, precipitation amounts and intensity may render the soil more vulnerable to erosion and increase slope instability.

**Table 3:** Annual and seasonal precipitation concentration index (PCI %) of the study area in the period of 1991–2021 years.

Districts		Precipitation Concentration Index (PCI %)	
	Annual	*Kiremt* (June–September)	*Belg* (February–May)
Babile	15.70	29.96	43.09
Chinakesen	15.47	28.52	41.39
Kombolcha	17.63	30.17	39.66
Haramaya	18.63	32.13	39.66
Goro gutu	17.71	31.44	38.19
Kurfa chale	14.62	30.14	39.66
Mieso	14.62	27.71	37.38
Guba qoricha	14.22	30.71	48.08
Boke	13.56	29.92	37.94
Gemechis	13.78	30.51	37.51
Mesela	14.15	26.71	37.49
Daro lebu	14.17	30.99	40.54

For most districts, belg seasonal PCI values were 48.08, 43.09, 41.39, and 40.54% for Guba Koricha, Babile, Chinakesen, and Daro lebu, respectively (Table 3). This findings confirmed with [105] in particular area, the vulnerability of soil to erosion will affect the growth conditions of plants and agriculture practices, altering land-use management strategies and slope instability increase the losses of economic and life. This means that the PCI value has exceeded 20, indicating a remarkably irregular rainfall pattern throughout the year. The results aligned with the findings of [40,106]; Zamani et al., 2018) proposed need to analyze precipitation variability at different scales to address flood and drought events, ensure sustainable water resource management, assess rainfall's erosive effects on soil erosion, and suggest informed conservation measures.

### 3.4 Standardized rainfall anomaly index estimation model

**Fig. 5:**
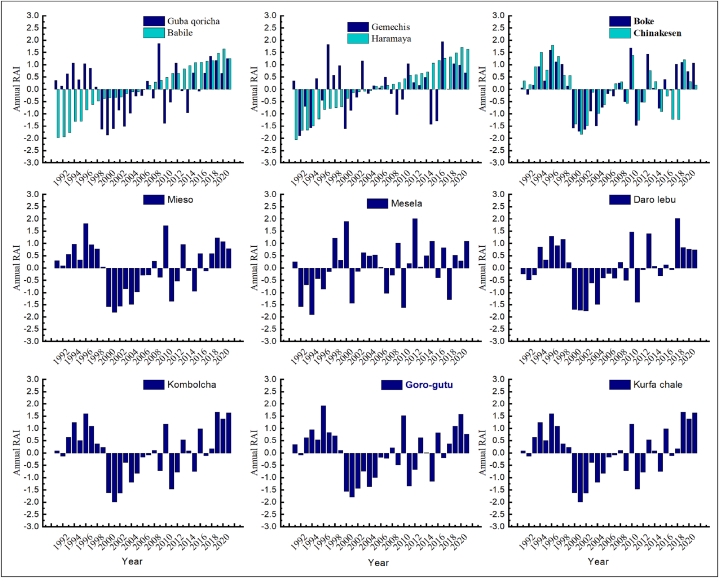
Annual rainfall anomaly index of weather station in districts of hararghe zone under the period of 1991–2021years.

**Fig. 6:**
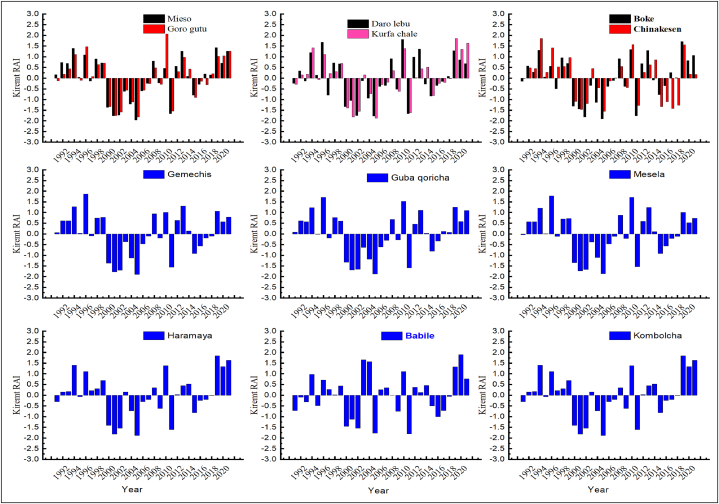
*Kiremt* seasonal rainfall anomaly index of weather station in districts of hararghe zone under the period of 1991–2021years.

**Fig. 7:**
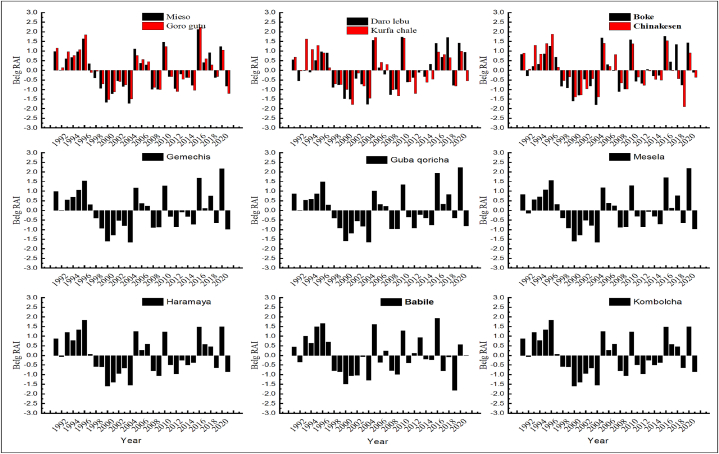
*Belg* seasonal rainfall anomaly index of weather station in districts of hararghe zone under the period of 1991–2021years.

Fig. 5, Fig. 6, Fig. 7 depicted that, annual, kiremt and belg rainfall anomaly index was calculated for the period from 1991 to 2021 years. In addition, the annual rainfall anomaly index for Goro gutu, Chinakesen, Haramaya, Kombolcha, Kurufa Challe and Babile, Mieso, Gemechis were moderately wet weather conditions during the period of 1997 (1.93), 1997 (1.79), 2020 (1.66), 2021 (1.65), 1997 (1.81), 2018 (1.94), respectively (Fig. 5). As a result, the annual precipitation anomaly indices for Mesela and Daro Lebu in 2013 (2.01) and 2019 (2.03) were very wet, respectively. However, Babile, Kombolcha, Kurfa Chale, Gemechis, Mesela, Guba Qoricha, Mieso in 1991, 2002, 2002, 1992, 1992, 1994, 2001, 2002 and Haramaya in 2002 (−2.04) were very dry conditions in the district from the eastern Hararghe zone (Fig. 5). This study shows that the kiremt indices for the seasonal precipitation anomalies at Chinakesen, Haramaya, Kombolcha, Kurfa Chale, Babile, Gemechis, Daro lebu, Boke and Mesela were very close to the regions of the dry season anomaly class of 1.99 climatic conditions of moderately wet weather from a regional study (Fig. 6).

### 3.5 Variability and trend analysis of rainfall and temperature characteristics of the study area

Annual and seasonal mean and coefficients of variation of climatic parameters, especially precipitation, minimum and maximum temperature time-series data, were analysed using the Mann-Kendall trend test of several eastern and western Hararghe (Table 4). The present results showed that, across the selected districts, a highest average annual precipitation of 848.2 mm and a CV% value of 26.1%, and a significant increasing trend of 3.870-fold and a 0.518 sense slope were observed in the study area (Table 4). As a result, the annual precipitation gradients for Haramaya, Goro Gutu, Guba Koricha, Gemechis, and Daro lebu Sens slope increased by 2.103, 2.902, 5.801, 5.638, and 4.155 times, respectively (Table 4). However, trend analysis from the Chinakesen weather station showed a decrease in annual precipitation, kiremt and belg seasonal precipitation, which significantly reduced the annual sen's slope for this region by factors of −6.104, −1.663, and −2.367. Similarly, M − K tests for Haramaya, Goro-gutu, and Kurfa chale were trending down and not statistically significant at alpha values with a confidence level of 0.05. Climate indices, including the kiremt rainfall which are designed to capture and quantify specific aspects of climate variability and change, other climate indices that cover a broader range of variables may used. These indices incorporate multiple climate parameters like temperature, humidity, wind speed and atmospheric pressure to provide a more holistic understanding of climate condition. It considers precipitation patterns and characteristics during the kiremt (JJAS) season, which is the primary rainy season in Ethiopia.

**Table 4:** Variability and time series trend analysis of rainfall in the study area.

Districts	Mean			CV%			ZMK			Slope		
	Annual	kiremt	belg	*annual*	*kiremt*	*belg*	annual	kiremt	belg	*annual*	*kiremt*	*belg*
Babile	761.2	324.9	329.8	26.8	38.0	41.1	0.045	0.135	−0.037	1.596	2.633**	−1.067
Chinakese.	758.4	309.2	250.1	30.9	34.7	43.3	−0.140	−0.114	−0.114	−6.104**	−1.663	−2.367**
Kombolch.	848.2	451.6	269.6	27.9	26.1	41.5	0.42	0.092	0.028	3.870**	2.233**	0.518
Haramaya	808.8	541.6	31.6	29.4	25.6	27.5	1.71	0.522	−0.028	2.103*	4.233**	−0.488
Goro gutu	787.1	439.4	255.9	25.6	35.0	43.9	0.075	0.097	−0.062	2.902*	3.227***	−1.326
Kurfa chal.	713.4	451.6	269.6	33.3	27.6	43.5	0.071	0.092	−0.028	1.207	2.233**	−0.488
Mieso	615.8	410.9	286.4	28.6	34.2	35.5	0.124	0.053	0.024	0.081	1.296	0.13
Gubaqoric.	815.5	530.2	286.4	25.7	34.2	38.5	0.114	0.062	0.163	5.081**	1.296	0.41
Boke	611.4	476.9	290.7	25.3	32.0	35.2	0.187	0.057	0.105	0.063	1.875	2.025**
Gemechis	718.4	685.0	881.4	24.4	28.4	27.4	0.256	0.316	0.398	5.638**	2.889**	3.366***
Mesela	548.8	521.1	304.7	27.8	34.5	36.9	0.032	0.028	0.002	1.019	0.601	0.008
Daro lebu	740.4	380.9	220.0	26.0	33.4	39.8	0.222	0.080	0.174	4.155**	1.818	2.81**

Note: CV%, Coefficient of variation; Note: CV, Coefficient of variation; ZMK; Mann–Kendall trend test, slope (Sen's slope) is change/yr, and *, **, significant at 0.05 significance level.

In addition, trend analyses of annual kiremt and belg maximum temperatures in the study area show a slightly increasing trend, with the exception of Guba Qoricha, Boke, Daro Lebu, and Mieso's Mann-Kendall test, with an alpha of 0.05 confidence level. value was not statistically significant regional weather stations (Table 5). As shown in Table 5, the results of the MK trend test showed that the average minimum temperature increased significantly over time with a 95% (0.05) confidence level. In addition, kiremt precipitation MK trend tests for the Babile, Kurfa Chale, and Mieso districts increased by factors of 2.016, 2.043, and 2.117, respectively, with a 95% (0.05) confidence level for the period 1991–2021 was significant. level. Also, the Sen slopes for the districts of Babile, Kurfa Chale, and Mieso are statistically significant values of 3.026, 2.014 and 2.186 or 30.24, 20.14 and 21.86 per decade for harvest season rainfall in belg increases by a factor of (Table 5). The overall increase in annual minimum, kiremt minimum and belg minimum temperatures observed in the study area is attributed to an increase in minimum temperature (increase in minimum temperature is more pronounced than maximum temperature). This result is consistent with the findings of [[107], [108], [109], [110]]; Tabari, H., Talaee, P·H., 2011) the increasing trend of the Tmin series is higher than that of the Tmax series.

**Table 5:** Variability and time series trend analysis of temperature in the study area.

Maximum temperature												
Districts	Mean			CV%			ZMK			Slope		
	Annual	kiremt	belg	*annual*	*kiremt*	*belg*	annual	kiremt	belg	*annual*	*kiremt*	*belg*
Babile	29.0	26.8	30.6	2.6	3.9	3.4	0.135	0.015	0.153	0.019	0.004	0.024
Chinakese.	27.2	26.2	28.9	3.1	4.1	3.2	0.226	0.226	0.178	0.037	0.048	0.031
Kombolch.	30.7	28.5	31.4	2.8	3.6	2.6	0.054	0.071	0.067	0.007	0.014	0.009
Haramaya	26.9	25.5	29.4	2.8	3.6	2.9	0.054	0.071	0.067	0.007	0.014	0.009
Goro gutu	29.3	29.8	31.2	2.7	3.7	2.8	0.062	0.088	0.037	0.006	0.017	0.005
Kurfa chal.	27.9	27.5	29.4	2.8	3.6	2.3	0.054	0.071	0.067	0.007	0.014	0.009
Mieso	28.7	28.1	31.4	2.9	4.0	21.4	0.084	0.028	−0.045	−0.132	0.008	−0.007
Gubaqoric.	25.6	27.1	30.1	2.9	4.0	3.1	−0.024	0.38	−0.045	−0.002	0.180	−0.0412
Boke	28.4	27.6	29.7	3.1	4.0	3.3	−0.041	−0.041	−0.002	−0.006	−0.006	−0.0002
Gemechis	27.4	25.2	28.4	3.4	4.3	2.4	0.045	0.062	0.067	0.062	0.013	0.012
Mesela	29.2	26.2	28.9	3.4	4.3	3.5	0.075	0.462	0.016	0.012	0.131	0.41
Daro lebu	30.3	29.8	31.2	2.3	3.1	2.8	0.011	−0.011	−0.032	0.001	−0.002	−0.007
Minimum temperature												
Districts	Mean			CV%			ZMK			Slope		
	Annual	kiremt	belg	*annual*	*kiremt*	*belg*	annual	kiremt	belg	*annual*	*kiremt*	*belg*
Babile	16.35	15.6	16.4	2.4	3.3	3.8	0.346	0.191	0.295	0.02	2.016**	3.028**
Chinakese.	13.6	14.4	14.6	2.9	3.0	4.4	0.136	0.458	0.290	0.023	0.032	0.032
Kombolch.	15.2	16.0	16.1	2.3	2.6	3.3	0.178	0.290	0.252	0.013	0.023	0.024
Haramaya	13.2	16.0	16.1	2.3	2.6	3.3	0.178	0.290	0.252	0.013	0.023	0.024
Goro gutu	15.0	18.2	17.8	2.3	2.9	3.04	0.187	0.213	0.273	0.014	0.021	0.025
Kurfa chal.	16.4	16.0	16.1	2.3	2.6	3.3	0.128	0.290	0.252	0.011	2.043**	2.014**
Mieso	17.7	16.5	16.5	2.4	3.0	3.1	0.200	0.204	0.218	0.014	2.117**	2.186**
Gubaqoric.	15.7	16.5	16.5	2.4	3.0	3.1	0.200	0.164	0.308	0.014	1.108	1.028
Boke	16.7	15.5	16.1	2.5	2.8	3.2	0.170	0.243	0.299	0.013	1.019	1.126
Gemechis	12.6	13.1	28.9	5.7	3.5	3.5	0.032	0.045	0.252	0.006	0.003	0.029
Mesela	14.6	14.6	15.7	2.4	3.1	3.2	0.243	0.312	0.372	0.015	0.024	0.03
Daro lebu	16.6	16.6	17.7	2.4	3.4	2.4	0.178	0.178	0.247	0.013	0.016	0.024

Note: CV, Coefficient of variation; ZMK; Mann–Kendall trend test, slope (Sen's slope) is change/yr, and *, **, significant at 0.05 significance level.

## 4 Discussions

The minimum length of growing season (LGP) for Babile, Chinakesen and Mieso were 37, 32 and 33 DOY, while the maximum LGP for Haramaya, Daro lebu, Kombolcha and Guba koricha were 253, 247, 239 and 202 DOYs, each in over the area. This is because the longest growing season/season observed in the Haramaya area is due to its high altitude, and the rainfall that was observed in this area lasted until September, resulting in a shorter growing season for the crops. According to Refs. [111,112] assessed that the impact of climate change on agro-ecological characteristics by looking at changes in length of growing period (LGP), as an initial proxy for agricultural impacts. Changes in rainfall patterns, in addition to shifts in thermal regimes, influence local seasonal and annual water balances and in turn affect the distribution of periods during which temperature and moisture conditions permit agricultural crop production.

Length of growing season means have to wait longer to mature crop yield. In contrast, the growing season was short in the semi-arid areas of the eastern hararghe zone, especially in the Chinakesen area. This is due to the hotter-than-average climate for the region, which can have a significant impact on when crops reach maturity. The late start and erratic rainfall may have reduced yields of some crops, such as sorghum and maize production, which are heavily cultivated in the eastern and western hararghe zones. Current results are consistent with [113] showing that delaying the onset of the rainy season is the least desirable practice as it shortens the growing season and reduces the plant's ability to meet its water needs. For these reasons, farmers may decide that it is best to change the planting date and the length of the rainfall start period in the study area to increase yield. The minimum and maximum or the earliest and latest start (onset date) of the rainy season in Kombolcha and Haramaya were 81 DOY (21 March) and 225 DOY (13 August), respectively. This result congruent with [91,114,115] revealed that, rainfall onset controls the best planting dates (when the soil moisture is sufficient to sustain the crop from germination to maturity), while the cessation and length of rainy season determine the type of seed to plant. Another study [116], who noted that, agricultural activities and production becames extremely vulnerable to the varaiability of the onset, cessation dates and length of the rainy season. After the end of the rainy season on September 1st, the moisture content of the soil becomes zero every day. This result was consistent with the findings of (Mulugojjam and Ferede, 2012) who showed that the soil moisture is zero at the end of the rainy season after September 1st, allowing harvest after the rains. The inter-annual variability of the begin of the wet season withinside the Haramaya district had a maximum coefficient of version of 38.6%, which became derived from a median begin of the wet season of approximately 125DOY recorded. The maximum profound issues related to rainfall variability because of modifications in moisture availability are: fairly variable date of wet season onset from one station to another, the temporal (month-to-month and annually) distribution of rainfall at every station or over a positive place and the ultimate is the cessation and duration of wet season. Hence, it's miles the vital to discover the rainfall and its spatial variability over the gap in conjunction with its duration of developing length to make crop primarily based totally decisions [117]. This suggests that the begin of the wet season is fairly variable withinside the place.

Estimated Sen slopes for start, end and LGP from 1991 to 2021 were calculated for 12 districts, while LGP for Gemechis district decreased by a factor of −2.533 which are highly significant at 0,05 confidence level. The dry season was a period of unusually long dry weather, shorter than a drought, less severe than a drought. This means that even during the main rainy season, a 5-day drought is very likely. Analysis of drought characteristics helped farmers effectively and efficiently control water supply for supplemental irrigation in key areas of long- and short-lived crops. The PCI values for seasonal precipitation for kiremt and belg in different districts of the eastern and western Hararghe zones range from 29% to 48%, especially in the belg seasonal precipitation pattern, where the monthly distribution of precipitation is significantly irregular indicates that in this area from February to May in the rainy season. This result was consistent with the findings of [118] that changes in precipitation concentration are associated with changes in atmospheric circulation patterns. Hence, over 70% of the precipitation fell in just three months from March to May in this study area.

The Goro Gutu kiremt RAI probably increased 2.06 times (2010) and is very wet in the selected areas. However, the belg rain anomaly index from Kurfa Chale, Mieso, Babile, Mesela, Guba Qoricha and Boke meteorological stations declined in moderately dry conditions, exposing the region to drought-prone areas. These results are consistent with those of [119] that drought is one of the most costly and complex natural. It has been shown to have significant and widespread adverse effects on many sectors that includes livestock and agricultural land. Kurfa chale belg coefficient of variation (CV%) 43.5 are higher than kiremt precipitation variability of 26.1 for the Kombolcha districts and the downward trend in belg precipitation is statistically decreasing. This means that the annual distribution of precipitation in belg is larger than the distribution of seasonal precipitation in the study area. The results was confirmed by the findings of [19,120,121]; Y [122,123]. revealed that a large part of Ethiopia finds greater variability in precipitation in belg than in kiremt.

Also, the Sen slopes for the districts of Babile, Kurfa Chale, and Mieso are statistically significant values of 3.026, 2.014 and 2.186 or 30.24, 20.14 and 21.86 per decade for harvest season rainfall in belg increases by a factor of. The overall increase in annual minimum, kiremt minimum and belg minimum temperatures observed in the study area is attributed to an increase in minimum temperature (increase in minimum temperature is more pronounced than maximum temperature). This result is consistent with the findings of [107,108,110]; Tabari, H., Talaee, P·H., 2011) the increasing trend of the Tmin series is higher than that of the Tmax series.

## 5 Conclusion and directions for future research

### 5.1 Conclusion

This study was examined climatic features, M − K trend test, variability, precipitation concentration index and temperature variability on the role of smallholder farmers in eastern Ethiopia. In this study, historical climate data was collected from daily weather stations of 12 districts were collected from Ethiopian National Meteorological Institute and compared with the national aeronautics and space administration or prediction of worldwide energy resources climate data. XLSTAT2018 statistical software was used for data quality checks, trend tests and outlier detection tests. Climate characterization was investigated using the climate guide software InStat +3.37.

In Kombolcha and Haramaya, the earliest and latest rainy seasons start date were 81 DOY (21 March) and 225 DOY (13 August), respectively, within the average of 130 DOY and 125 DOY. This suggests that smallholder farmers in the study area should adapt effectively and plant crops early, as some crop varieties may only mature for a short period of time. Irregular rainfall and late start dates may have reduced yields for some crops, but the largest and most prevalent crops are sorghum and maize, with smallholder farmers in the eastern and western hararghe zones. The minimum end date of the rainy season for Babile, Boke and Daro Lebu were 245 DOY (September 2nd), while the maximum of 339 DOY (December 5th) averaged 252, 149, and within 277 DOY. Chinakesen and Haramaya were found to have the shortest and longest growing season at 32 DOY and 253 DOY respectively. The LGS, coefficients of variation for Mieso and Chinakesen were 36.2% and 47.9%, respectively, indicating that they were highly vulnerable to climatic disasters such as drought. Gemechis District Sen's Slope LGP decreased by a factor of −2.533 and this is very significant with an alpha value of 0.05. Both the eastern and western districts of the hararghe zone have a 70% chance of experiencing a 5-day drought. This indicates a very high risk of a 5-day drought during the rainy season for the main crop.

The PCI values for seasonal precipitation for kiremt and belg in the eastern and western Hararghe zone districts ranged from 29% to 48%, indicating a marked asymmetry in the monthly precipitation distribution, especially in belg, where the seasonal precipitation pattern is the strongest increase. The annual rainfall anomaly index for Mesela and Daro Lebu showed were consistently very wet in 2013 (2.01) and 2019 (2.03) respectively. In the Kombolcha and Kurfa Chale districts, the CV% were 41.5 and 43.5, respectively, which were higher than the kiremt rainfall variability of 26.1 and 27.6. Annual and belg seasonal rainfall of sen's slopes from the Chinakesen weather station decreased significantly by factors of −6.104 and −2.367.

### 5.2 Directions for future research

Based on the research findings and conclusion of the study the following recommendation was forwarded to further studies. The characterization of local climate is crucial, especially during the small rainy season (February to May) and the main rainy season (June to September), particularly for smallholder farmers in eastern Ethiopia. These farmers face challenges like limited land availability and reliance on dryland farming. The irregularity of rainfall patterns makes it extremely difficult to cultivate both long and short maturity crops. For example, this year saw significant rainfall from March 20 to June 8, followed by a prolonged dry spell from July to August. July, which is usually a critical period for crop flowering and maturation, experienced an unusual absence of rainfall.

➢ A climate characterization study was conducted on 12 selected stations in the East and West Hararghe zones. The onset and cessation dates of the rainy season in the eastern Hararghe zone were more appropriate compared to the west Hararghe zone due to the topography of the area, which is closer to highland areas like Haramaya and Kombolcha, known for their productivity in crop production.
➢ Precipitation concentration index (PCI) is an essential feature for water resources planning, prediction of risk due to droughts or floods and the management of natural resources.
➢ Realising strategies to address dry spells in the arid and semi-arid areas of Eastern Hararghe, such as cultivating sorghum and maize crop varieties with long and short maturity periods respectively, can be a beneficial solution for smallholder farmers. These crop varieties are not only easily accessible to farmers but also contribute to improved soil fertility status.
➢ There is a lack of comprehension regarding the characterization of climate which may result in difficulties for farmers in the study area, particularly during the onset and cessation of the rainy season.
➢ The study reveals a limited amount of research conducted on climate outlooks for providing agrometeorological advice to farmers.
➢ There exists a knowledge gap between the study of climate variability and the awareness of drought-tolerant crops among smallholder farmers in the study area.
➢ The quality control of climate information-based data, as well as the techniques used for data collection, were significant issues encountered during data recording and acquisition.
➢ This study suggests further research on examining different the climate extreme indices and identifying adaptation strategies that mimic climate change impacts and variability on smallholder farmers in the study area.

## Author contribution statement

Girma Asefa Bogale: Conceived and designed the experiments; Performed the data curation; Analysed and interpreted the data; Wrote the original paper.

## Data availability

The data supporting the conclusions of the study can be accessed through email to corresponding author.

## Funding

The authors did not receive any funds for this manuscript.

## Declaration of competing interest

The authors declare that they have no known competing financial interests or personal relationships that could have appeared to influence the work reported in this paper.
